# Babies before business: protecting the integrity of health professionals from institutional conflict of interest

**DOI:** 10.1136/bmjgh-2022-009640

**Published:** 2022-08-04

**Authors:** Genevieve Ellen Becker, Constance Ching, Tuan T Nguyen, Jennifer Cashin, Paul Zambrano, Roger Mathisen

**Affiliations:** 1 BEST Services, Galway, Ireland; 2 Alive & Thrive Southeast Asia, FHI Solutions / FHI 360, Washington, District of Columbia, USA; 3 Alive & Thrive Southeast Asia, FHI Solutions / FHI 360, Hanoi, Viet Nam; 4 Alive & Thrive Southeast Asia, FHI Solutions / FHI 360, Manila, Philippines

**Keywords:** Child health, Health policy, Health systems, Public Health, Maternal health

**Summary box**

- The commercial milk formula industry’s duty to maximise profits conflicts with the health system’s duty to protect health and to support breastfeeding.
- The marketing tactics and relationships with the commercial milk formula industry including financial or material support, sponsorship of training or research and advertising in journals or at events contribute to conflicts of interest within the health system.
- Employers of health workers, academic institutions, professional associations and governments all have a duty of care to protect health systems from predatory marketing and to facilitate individual health workers to practice in an ethical manner.
- National regulatory measures to implement and ensure monitoring of compliance with the International Code of Marketing of Breast-milk Substitutes protect health workers in addition to children and their families.

Manufacturers and distributors of commercial milk formula (CMF), or breast milk substitutes (BMS), a US$ 55 billion industry,^1^ have a duty to their shareholders to maximise sales. Marketing increases CMF sales—but reduces breastfeeding. The health system and those who work within it have a primary obligation to preserve and improve health outcomes. Fulfilling this obligation requires that breastfeeding is protected, supported and promoted. These two interests—maximising CMF sales and protecting, supporting and promoting breastfeeding—directly conflict with each other. Conflicts of interest (COI) arise within practices such as sponsorship and funding that bind companies and health systems together.^2^ In these situations, professional judgement concerning a primary interest (unequivocal support for breastfeeding) tends to be unduly influenced by a secondary interest (sponsorship by or partnership with industry).^3^ This conflict is even more evident when CMF marketing targets the health system itself.

Infant and young child feeding (IYCF) practices have lifelong effects on the child, the mother, the family, the wider community and on environmental sustainability. As highlighted in the recent report from the World Health Organization (WHO) and United Nations Children’s Fund (UNICEF),^1^ health systems and health workers have significant influence on decisions and practices related to IYCF and child care. CMF companies understand the influence of health workers on feeding decisions and consequently focus marketing efforts on those responsible for health policies and practices—service managers, health workers and their professional associations, researchers and academic institutions.^4–7^ Their many and varied marketing tactics include providing financial support to attend conferences, funding conferences, providing education sessions, funding research, donating low-cost supplies of CMF to health services and in emergency situations, donating equipment and providing IYCF ‘education’ to parents via the health system, among others.^8–11^

These approaches create conflicts for the health system and health workers influencing them to act in ways that impede fulfilling their ethical obligations, compromise professional judgement, integrity and public credibility towards their protection of breastfeeding, at both individual and institutional levels.^2 12^ However, some health professionals still hold a view that sponsorship of educational events and partnerships can be managed in a manner that is more lenient than the WHO guidance.^13^

For example, arrangements between a health service and a CMF company to use a specific brand may create expectations that health workers will give samples of specific products to all new parents. Similar pressure occurs when health workers attend events that are sponsored by the companies which influence who speaks or what content is presented and what products are exhibited at these events, or when companies sponsor health workers to attend conferences. The recent commentary by Pereria-Kotze *et al*^14^ showed how scientific and professional journals may be led by associations or individuals that receive funding and may thus act as a conduit for marketing of products which may directly conflict with public health guidance.

The dangers of this marketing have been recognised for decades. In 1981, the International Code of Marketing of Breast-milk Substitutes (the Code)^15^ was adopted to protect infants and young children from harmful marketing. It can also protect health workers, the health systems they work in and the academic institutions that educate them from marketing that creates COI. The Code states that no financial or material inducements by manufacturers or distributors should be offered to or accepted by health workers. The World Health Assembly (WHA) developed more detailed guidance^16^ for both the CMF industry and health workers to prevent COI. Implementation of the Code, the subsequent WHA resolutions and the Baby Friendly Hospital Initiative guidelines^17^ support health workers to practice free of the influence of commercial marketing. 

Yet four decades since the adoption of the Code, these marketing practices persist.

A recent review was the first systematic scoping of published global research documenting evidence of Code violations from 1981 to August 2021.^18^ Of the 153 articles reviewed, 28 studies documented practices involving COI as outlined in relevant Code provisions (Article 7) and WHA resolutions (WHA resolution 49.15 (1994), WHA resolution 58.32 (2005) and WHA 69.9 (2016)/guidance).^19^ Two-thirds of the COI findings were published between 2010 and 2021.

In the 28 studies documenting potential COI, this was reported most frequently in hospitals and in non-hospital health facilities, and then in medical schools or universities (figure 1). The products marketed included infant formula, ‘follow-on’ and ‘growing up’ milks and complementary foods, as well as bottles, teats and pacifiers or marketing using a brand name encompassing a range of products. Marketing activities included financial and other incentives to health workers (21 studies), companies providing education on IYCF to parents through health facilities (15 studies), sponsorship of health workers’ meetings and conferences (14 studies), scholarships to health workers (five studies) and using health facilities to host events for health workers (three studies) (figure 1). Some studies reported on multiple means of marketing. Thematic analysis of the 28 studies identified six major themes on COI: financial or material support, funding research, sponsorship of professional events, advertising in journals, sponsorship of breastfeeding activities and partnerships with governments (table 1).

**Figure 1:**
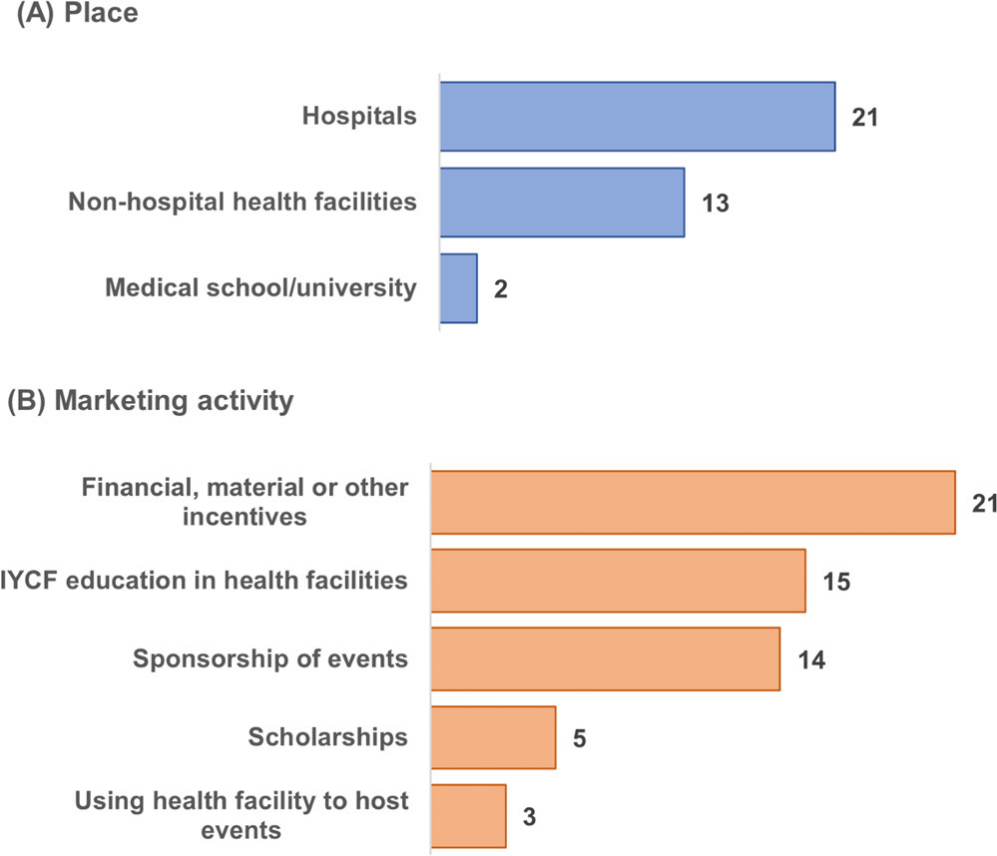
The number of studies in scoping review^18^ that documented practices that may result in conflicts of interests by place (A) and type of marketing activity (B). IYCF, infant and young child feeding.

**Table 1:** Types of COI involving CMF companies—examples from scoping review^18^

COI themes		Examples from scoping review^18^
1	Financial or material support to health workers, facilities and training establishments	Health workers in Pakistan received gifts labelled with CMF company names or sponsorship for conferences or training.*
		CMF companies funded professional development activities in the Americas, Asia and Europe.†
2	Funding of medical research	Nestlé sponsored research on hospitalised pre-term infants in India.‡
3	Sponsorship of professional associations: events and generic financial support	CMF companies sponsored publications and websites in Africa, the Americas, Asia and Europe.†
		A Royal College in the UK responsible for setting infant feeding policy and guidelines accepted funding from industry for activities related to ‘specialist’ formula.§
		More than 90 food industry actors sponsored 88% of nutrition conferences in Latin America and the Caribbean between January 2018 and December 2019. Abbott and Nestlé were the most frequent sponsors.¶
4	Advertising CMF products in professional journals	Neolacta Life Sciences, a BMS company, advertised their infant formula in the Journal of Neonatology, the official journal of the National Neonatology Forum of India.‡
5	Sponsorship of breastfeeding promotion and support activities	Nestlé sponsored the Kartini Program in Indonesia, a government programme to train midwives to support mothers on exclusive breastfeeding.**
6	Forging partnerships with governments	Danone distributed CMF through a partnership with the Central Java government of Indonesia during the COVID-19 pandemic. ††

*Salasibew M, Kiani A, Faragher B, Garner P. Awareness and reported violations of the WHO International Code and Pakistan's national breastfeeding legislation; a descriptive cross-sectional survey. International Breastfeed Journal 2008;3(24).

†Grummer-Strawn LM, Holliday F, Jungo KT, Rollins N. Sponsorship of national and regional professional paediatrics associations by companies that make breast-milk substitutes: evidence from a review of official websites. BMJ Open. 2019;9(8):e029035.

‡Gupta A. Under Attack: A report of the monitoring the compliance with the Infant milk substitutes, Feeding bottles and Infant foods (Regulation of Production, Supply and Distribution) Act 1992 and the Amendment Act 2003. Breastfeeding Promotion Network of India (BPNI); 2021.

§Hastings G, Angus K, Eadie D, Hunt K. Selling second best: how infant formula marketing works. Globalization and Health. 2020;16(1):77.

¶Mialon M, Jaramillo Á, Caro P, Flores M, González L, Gutierrez-Gómez Y, et al. Involvement of the food industry in nutrition conferences in Latin America and the Caribbean. Public health nutrition. 2021;24(6):1559-65.

**IBFAN-ICDC. (2017). Breaking the Rules (BTR), Stretching the Rules 2017: Evidence of violations of the International Code of Marketing of Breastmilk Substitutes and subsequent resolutions, compiled from June 2014 to June 2017. IBFAN. http://www.babymilkaction.org/wp-content/uploads/2021/04/2017-BTR-2017sm.pdf

††Ching C, Zambrano P, Nguyen T, Tharaney M, Zafimanjaka M, Mathisen R. Old Tricks, New Opportunities: How Companies Violate the International Code of Marketing of Breast-Milk Substitutes and Undermine Maternal and Child Health during the COVID-19 Pandemic. International Journal of Environmental Research and Public Health. 2021;18:2381.

CMF, commercial milk formula; COI, conflict of interest.

While health professionals may believe that these ‘gift relationships’ do not significantly compromise their professional judgement or create expectations or obligations, studies show otherwise.^2 7^ The impulse to reciprocate, even when gifts are of minimal value, influences objectivity and causes health workers to reweigh information and choices due to the indebtedness to the gift-givers, sponsors and partners.

One of the most effective and insidious ways to forge links with health workers is to contribute to their professional development.^20^ Very recent examples (from 2022) include sponsorship of the British Journal of Midwifery conference by two major CMF companies, Kendamil and Nutricia,^21^ and infant nutrition research funded and published by FrieslandCampina, a CMF manufacturer.^22^ The professional independence in these situations is inarguably compromised.

Even when companies are seemingly supporting breastfeeding promotion through programmes, informational materials or partnership with governments, the inherent COI creates questions for the health system. For example, distribution of sponsored breastfeeding informational materials may be in return for funding and these materials may over-emphasise breastfeeding difficulties and contain inadequate information, thus undermining successful breast feeding.^8^

Knowing the limited resources available to health facilities, companies provide equipment that is branded with their logo (a common marketing tactic),^20^ which could result in entrenched dependency that perpetuates the norm of accepting financial or material support.

Even among countries that adopted the Code, protection against COI is lacking in most despite the significant documented COI.^11^ Some professional associations have stopped taking funding from CMF companies^23^ as have some journals which previously carried marketing for these companies.^24^

Despite being aware of their Code violations and how these create problems for countries, associations and individuals, the CMF industry continues to use health systems to market its products, putting their commercial profits above the health and well-being of children, parents and health workers.

The scoping review^18^ of evidence of Code violations from 1981 to mid-2021 adds to the mounting global evidence on the magnitude of the problem of COI in the marketing of CMF. Clearly, the issue of COI is not new—and it must not be allowed to persist.

We, therefore, urge all governments to protect the integrity of the health system and its workers by adopting all COI safeguards put forth in the Code and relevant WHA resolutions. We call on associations of health professionals and of students, education and accreditation bodies, health facilities and private health providers, to formally reject all forms of industry support and to adopt COI governing documents and enforceable codes of conduct.

Health service management guidelines and policy, using the Code and relevant WHA resolutions as a framework, must be developed and implemented to provide guidance in identifying and resolving COI situations. Training for health workers and officials should sensitise them to the risks of undue industry influence on their duty to protect health.

If health systems and health workers are to provide an environment of care that is ethical and reflects best practice in supporting good health and nutrition for infants, young children and their mothers, then there is an obligation to protect the health workers from being profoundly undermined by the institutional conflict of interest. This marketing of CMF within the health system needs to stop.

## Data Availability

There are no data in this work. As this article is based on a systematic review of existing data and no new data was collected, data sharing is not applicable to this article.
